# Sorting and Transporting a Viral Cargo: The Role of the Rab7 Protein

**DOI:** 10.1371/journal.pbio.0030260

**Published:** 2005-06-21

When Robert Hooke first looked at cork bark with a light microscope in 1655, he saw small empty chambers, reminiscent of monastery cells. We now know that living cells are full of organelles—specialized subcompartments surrounded by membranes in which different cellular life functions occur. This complex organization raises major transport and sorting problems similar to those encountered in a large city in which trains and trucks carrying different cargos arrive at peripheral distribution centers. The cargos must be sorted and transported to individual factories where goods are made for delivery to other city destinations or for export. At the same time, the different areas of the city produce waste products that also need to be sorted and transported correctly. Somehow, thousands of cargos must end up in exactly the right place in both the city and the cell.

One cellular system that sorts and transports cargos is the clathrin-mediated endocytic pathway. Endocytosis—the ingestion of materials into the cell—is important for the interaction of cells with the environment because it allows the uptake of nutrients (the equivalent of the raw materials brought into the city) and signaling molecules (the letters brought in by the mail service). In clathrin-mediated endocytosis, materials arriving at the outside surface of the cell are engulfed in special areas of membrane known as coated pits, which pinch off to form intracellular vesicles. These lose their clathrin coat and other molecules involved in their formation to become early endosomes, a specific sort of intracellular vesicle. The cargos are then transferred to late endosomes, which have different proteins and functions than early endosomes. From endosomes, cargo can go either to lysosomes, where they are degraded, or to the Golgi apparatus, which sends cargo back to the cell surface.

Although many details of clathrin-mediated endocytosis have been uncovered, cell biologists still hotly debate whether early endosomes mature into late endosomes or whether transport vesicles take cargos from early to late endosomes. Unraveling such details will improve our understanding of normal cellular processes and should help in the design of intracellularly targeted drugs. Andreas Vonderheit and Ari Helenius now provide new insights into this controversy by examining how Semliki forest virus (SFV) is sorted and transported to late endosomes. [Fig pbio-0030260-g001]

**Figure pbio-0030260-g001:**
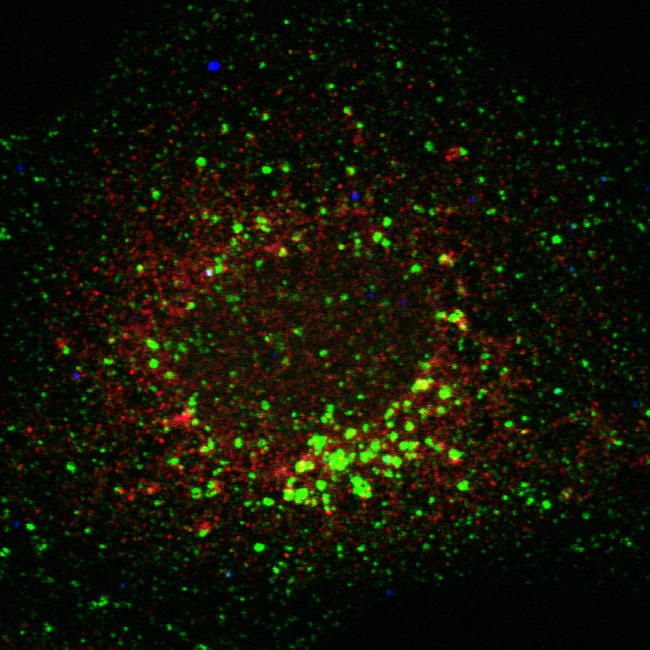
Immunofluorescence showing intracellular compartments containing Rab7 (red), EEA1 (green), and Semliki Forest Virus (blue)

Like many animal viruses, SFV enters its host cells using clathrin-mediated endocytosis. One well-established way to study this process is to attach a fluorescent tag to individual virus particles and observe their travels through the cell. Vonderheit and Helenius now track this journey in greater detail than ever before by attaching different colored fluorescent tags to SFV and to protein markers of early and late endosomes. They then use video-enhanced triple-color microscopy to follow all the markers as they move through living cells. This analysis reveals that the virus is initially present in endosomes containing only proteins associated with early endosomes. Then, Rab7, a late endosome marker that is involved in transport of cargo from early to late endosomes, appears in distinct domains of these early endosomes. Finally, the viral cargo is transferred to a detached organelle that contains Rab7 but no early endosome markers. The researchers show that SFV transport to late endosomes requires Rab7 and the presence of intact microtubules, which often serve as a highway network along which vesicles travel.

The researchers conclude that, at least for SFV, the mechanism underlying sorting and transport from early to late endosomes falls somewhere in between the two existing models for clathrin-mediated endocytosis. Early endosomes, they postulate, have to acquire some characteristics of late endosomes before SFV can be transported to late endosomes in Rab7-positive vesicles. But other cargos, the authors point out, may follow different pathways through the cell.

